# A Three-Dimensional Atlas of the Honeybee Neck

**DOI:** 10.1371/journal.pone.0010771

**Published:** 2010-05-24

**Authors:** Richard P. Berry, Michael R. Ibbotson

**Affiliations:** ARC Centre of Excellence in Vision Science, Division of Biomedical Science and Biochemistry, School of Biology, Australian National University, Canberra, Australian Capital Territory, Australia; Centre de Recherches sur la Cognition Animale - Centre National de la Recherche Scientifique and Université Paul Sabatier, France

## Abstract

Three-dimensional digital atlases are rapidly becoming indispensible in modern biology. We used serial sectioning combined with manual registration and segmentation of images to develop a comprehensive and detailed three-dimensional atlas of the honeybee head-neck system. This interactive atlas includes skeletal structures of the head and prothorax, the neck musculature, and the nervous system. The scope and resolution of the model exceeds atlases previously developed on similar sized animals, and the interactive nature of the model provides a far more accessible means of interpreting and comprehending insect anatomy and neuroanatomy.

## Introduction

Until recently, the fields of anatomy and histology have relied on dissection and description, drawings, and sectioning of specimens. These approaches are limited by an inability to describe a structure in three dimensions. Two-dimensional description, even from multiple points of view, is often insufficient to relay the depth and complexity of a biological three-dimensional (3D) structure. Microscopes and imaging techniques are now sufficiently advanced to allow large scale collection and analysis of 3D data [1]–[6]. These approaches have led to the generation of digital 3D atlases of various structures, e.g. rat [7], human [8], and insect brains [4], [9].

Digital 3D atlases have a number of advantages. Digital models can be easily viewed from many directions, providing an intuitive 3D representation. The ability for the user to interact with a 3D model greatly facilitates the learning process. Digital models can also be easily disseminated to interested parties, and steadily enhanced over a period of time.

For these reasons, we undertook the development of a comprehensive 3D model of the honeybee neck. The head-neck system is of particular interest in insects (which have fixed eyes), because the entire head must be stabilised during movement to overcome visual problems, such as disambiguating rotational from translational optic flow [10], [11]. We chose the honeybee because it is one of the organisms commonly taken as being representative of flying insects. The morphology of the honeybee neck has been described on three previous occasions. Snodgrass [12] described the entire “miniature machine”, including the skeletal structure and musculature of the neck. Markl [13] described the skeletal structure, musculature and innervation pattern of the thorax. Schröter et al. [14] described the morphology of two of the neck muscles in detail, and also identified individual innervating motor neurons.

While these studies are well documented and illustrated, they suffer from an inability to describe complex 3D information, due to the two-dimensional mode of presentation. In the present study we address this issue by providing a 3D digital atlas of the honeybee neck. In generating the atlas we aimed to: validate the morphological descriptions of earlier studies; provide an accurate representation of external and internal prothorax morphology in an easily interpretable format; and use the 3D model as a tool for predicting muscle function.

## Results

**Figure 1:**
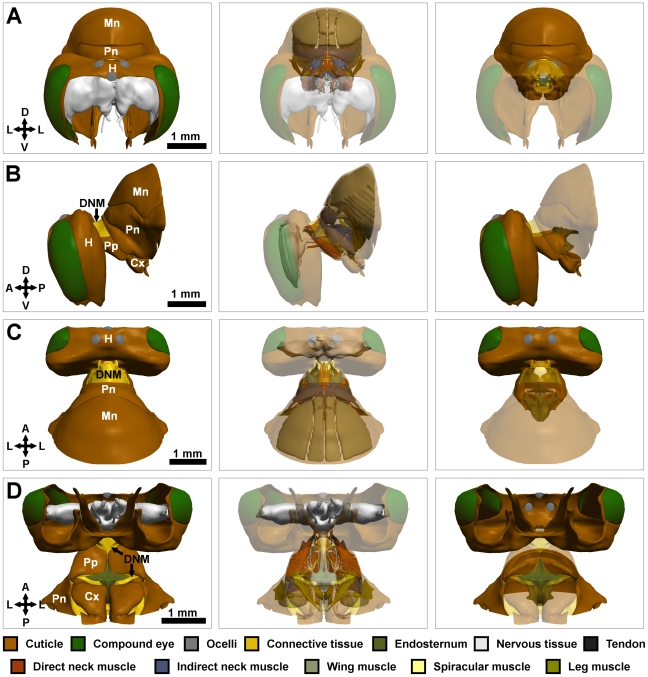
Orthogonal views of the reconstructed model. Left column: model with no translucency. Middle column: model with exoskeleton translucent. Right column: only exo- and endo-skeletal components shown. Surface structures partially translucent. (A) Frontal view (minus frons and mouthparts). (B) Side view. (C) Dorsal view. (D) Ventral view. Cx: coxa of the foreleg; DNM: dorsal neck membrane; H: head; Pn: pronotum; Pp: propectus; Mn: mesonotum. D: dorsal; V: ventral; L: lateral; A: anterior; P: posterior.

The complete interactive model is available for download (Fig. S1). For orientation purposes, Fig. 1 shows orthogonal views of the model. Table 1 provides an overview of the attachment points of each of the neck muscles, the innervating nerves, the probable functions of the muscles, and any points of contention with previous studies. Except where otherwise indicated, the terminology used arises from Snodgrass [12].

**Table 1:** Comparison of gross muscle morphology and function across three studies.

Type	Name	Insertion	Origin	Innervation	Function	Snodgrass [12]	Markl [13]
**Direct neck muscle**	40a	SA (lateral) *	Pph (lateral)*	IK2	Head levator/abductor	* Not described	As described here
	40b	Same as 40a	PPI (medial)*	IK2	Head levator/rotator**+**	* Prephragma (medial)+Head levator	As described here
	41a	FM (medial)*	Pph (lateral)*	IK2	Head levator/lateral translator	* Not described	As described here
	41b	Same as 41a	Pph (medial)	IK2	Head levator	As described here	As described here
	42a	SA (apical)	HA	IN1	Head levator/abductor**+**	**+**Head levator/rotator	As described here
	42b	Same as 42a	Pp (pleural)	IN1	Head rotator/levator	As described here	As described here
	42c	Same as 42a	Pp (sternal)	IN1	Head rotator/levator	As described here	As described here
	43	SA (lateral)	SNBE (lateral & medial)	IN1	Head levator/abductor**+**	**+**Head levator	As described here
	44	SC	SNBE (anterior)	IK1	Head depressor	As described here	As described here
**Indirect neck muscle**	45	Pn (medial)	Pph (medial)	IK2	Pn retractor**+**	+Pn depressor/Mn retractor	As described here
	46	CA (medial)	Pn (lateral)*	IK2	OP levator/abductor/rotator**+**	* Pph (lateral)**+**Not described	As described here
	47	Same as 46	PPI (medial)	IK2^▴^	OP levator**+**	**+**Not described	^▴^ Not described
	48	HA (anterior)	PPI (medial)	IN1/IK2	OP levator/adductor/rotator**+**	**+**Not described	As described here
	49	API (anterior)	PA	IN1	OP protractor	As described here	As described here
	50	API (posterior)	PA	IN1	OP depressor/protractor**+**	+OP protractor	As described here
	51a/51b	CA	SNAE	IK1	OP retractor/adductor**+**	**+**OP adductor	As described here

Overview of gross morphology, innervation and function of the neck musculature as described here, by Snodgrass [12] and by Markl [13]. API: Anterior pronotal inflection; CA: cervical apodeme of propectus; HA: Horizontal apodeme of the propectus; Mn: Mesonotum; FM: Rim of foramen magnum; OP: Occipital process; PA: pleural apophysis of propectus; Pph: Prephragma of mesonotum; PPI: posterior pronotal inflection; Pn: Pronotum; Pp: propectus; SA: Supraforaminal apodeme of the head; SC: Subforaminal cup; SNAE: Supraneural apodeme of endosternum; SNBE: Supraneural bridge of endosternum. Symbols: (*) and (^▴^) indicate that the anatomy or innervation of a muscle described here differs to that described by Snodgrass [12] and Markl [13] respectively; (+) indicates that the function of a given muscle is considered to be different to that described by Snodgrass [12].

### Head structure

**Figure 2:**
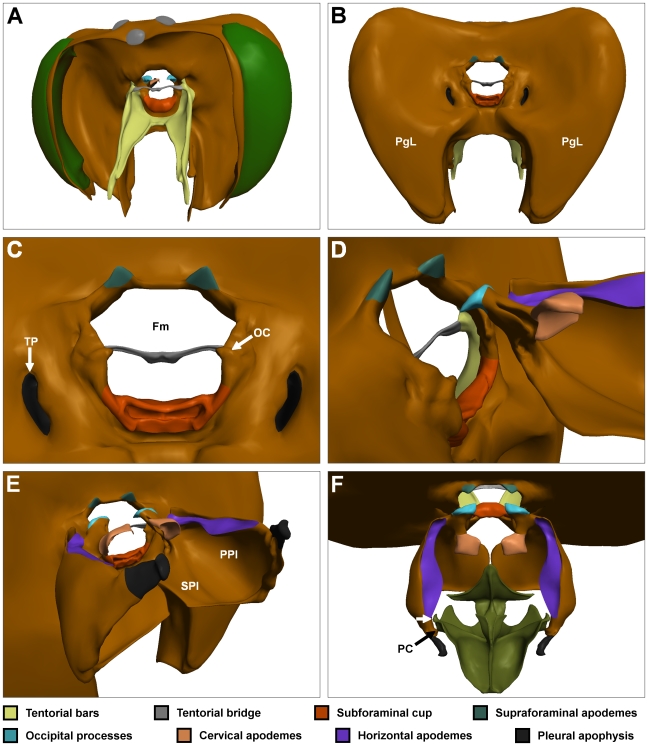
The head and propectuses. (A) Oblique frontal view. (B) Posterior view of the head. (C) Enlarged view of the foramen magnum and surrounding structures. (D) Articulation of the right propectus with the head (left propectus not shown). (E) Posterolateral view. (F) Dorsal view including the endosternum. White arrow marks a protruding flange that may prevent over-protraction of the propectus. FM: foramen magnum; OC: occipital condyles; PC; propectal condyle; PGL: postgenal lobes; PPl: pleural plate of propectus; SPl: sternal plate of prospectus; TP: tentorial pits. Colour codes as in Fig. 1.

The weight of the head is borne by the direct neck muscles, which insert onto a thickened ridge of cuticle surrounding the foramen magnum. Around this ridge, a number of thickened foldings of the head cuticle are present (illustrated in colour code in Fig. 2). Of interest are the two *supraforaminal apodemes* (that attach to several neck muscles); the *occipital condyles*, upon which the pivot points of the prothorax sit; and the *subforaminal cup* (that attaches to the direct neck depressor muscle).

### Propectus

The *dorsal neck membrane* forms a cone of flexible connective tissue that connects the foramen magnum of the head to the cuticular structures of the prothorax (Fig. 1B, C). The only rigid tissues directly connecting the head and neck (the *occipital processes*) project inwards into the foramen magnum and articulate with the occipital condyles of the head (Fig. 2C, 2D). The occipital processes are the most anterior projections of a pair of bilaterally symmetrical structures, termed the *propectuses*. Each propectus is a section of cuticle containing a number of infoldings and protrusions, including the *sternal* (ventral) plate, *pleural* (lateral) plate, *horizontal apodeme*, *cervical apodeme* and *pleural apophysis* (Fig. 2). Each of these structures serves as the attachment site for one or more muscles. The sternal plates of the two propectuses run towards the midline but do not unite (Fig. 2F); flexible connective tissue joins the medial margin of each sternal plate to the overlying endosternum. A small medially projecting *propectal condyle* marks the site of articulation between the propectus and endosternum (Fig. 2F), which are bound by a strong tendon.

### Endosternum

**Figure 3:**
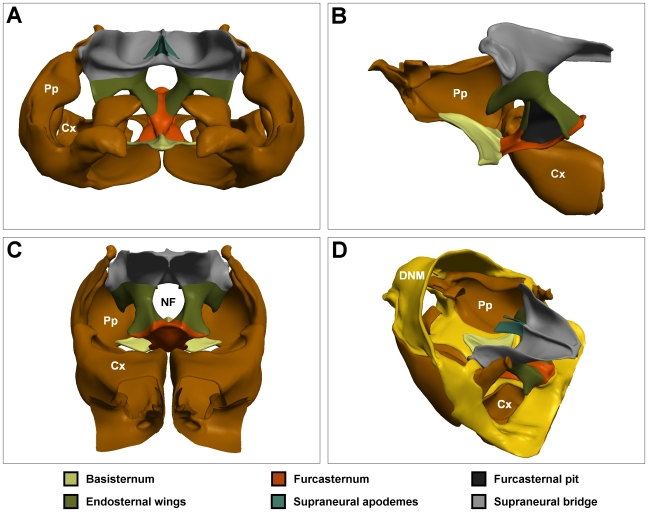
The endosternum. (A) Frontal view of endosternum and propectus. (B) Lateral view of endosternum, right propectus and right fore coxa. (C) Posterior view of endosternum, propectuses and fore coxae. (D) Oblique posterodorsal view of endosternum, propectuses, coxae and dorsal neck membrane. NF: neural foramen; Colour codes and labels as in Fig. 1.

Overlying the sternal plates of the propectuses is the *endosternum*. Ventrally, the basal surface of the endosternum forms part of the protective exoskeleton, interfacing anteriorly with the propectuses, and posteriorly with the forelegs. The basal plates provide solid foundations for large upward extending infoldings that provide protective housing for the nerve cord, and attachment points for several muscles of the neck and forelegs. The endosternum is a continuous structure that can be regionalized as follows (Fig. 3): the *basisternum*, the ventral protective plate; the *furcasternum*, which articulates with the coxae of the forelegs; the *endosternal wings*, which envelop the nerve cord; and the *furcasternal pits*, *supraneural bridge* and *supraneural apodemes*, which are all sites of muscle attachment.

### Pronotum

**Figure 4:**
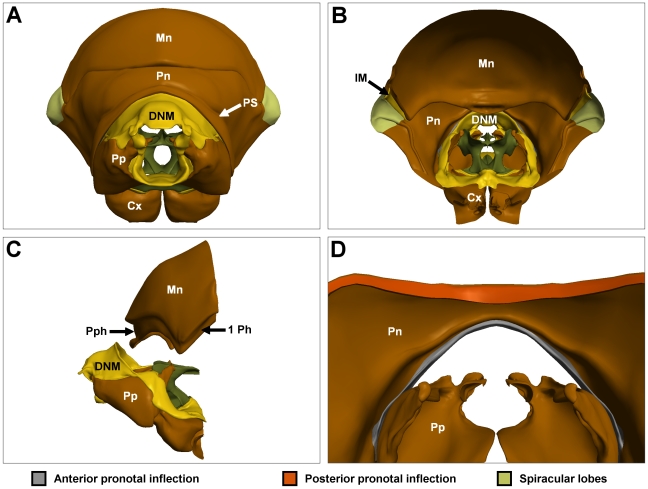
The pronotum, mesonotum and dorsal neck membrane (DNM). (A) Anterior view of skeletal structures of the prothorax. (B) Same as (A), posterior view. (C) Same as (A), lateral view. Pronotum not shown to expose the prephragma, first phragma and DNM. (D) Enlarged posterior view of pronotum and propectus. IM: intersegmental membrane; PPh: prephragma of the mesonotum; 1 Ph: first phragma of the mesonotum; PS: pronotal sulcus. Colour codes and labels as in Fig. 1.

The *pronotum* is the dorsal plate of the prothorax, forming a wide arc over the propectuses and encircling their pleural plates ventrally (Fig. 4A). Both the anterior and posterior margins of the pronotum are inflected to form the *anterior* and *posterior pronotal inflections* (Fig. 4D). These inflections serve as sites of muscle attachment, and also interface between the pronotum, the propectuses, and the mesonotum. Other structural features are the *pronotal sulcus*, a deep inflection visible externally, and the *spiracular lobes*, which form protective coverings over the first pair of spiracles (Fig. 4A). The reconstruction of the pronotum is not complete as it was beyond the scope of the present work.

### Mesonotum

The second notal plate, the *mesonotum*, serves two functions: externally it provides a protective housing for the mesothorax; internally it provides attachment points for several wing muscles (Fig. 1). Only the anterior region of the mesonotum is of interest here: the posterior mesonotum was not reconstructed. The most notable feature of the anterior mesonotum is the infolding of the body wall on the ventral rim (*prephragma* (anteriorly)) and *first phragma* (laterally)) (Fig. 4C). The anterior surface of the prephragma serves as an attachment point for several of the neck muscles, while the posterior surface serves as the ventral attachment point for wing muscle 71.

### Dorsal neck membrane

The large gap between the anterior margin of the pronotum and the foramen magnum of the head is bridged by a flexible membrane. Ventrally the membrane forms a cushioning surface between the occipital condyles of the head and the occipital processes of the propectuses. The membrane also links the sternal plate of each propectus to the head, thus forming a ring of soft tissue around the foramen magnum (Fig. 4A).

### Nervous system

**Figure 5:**
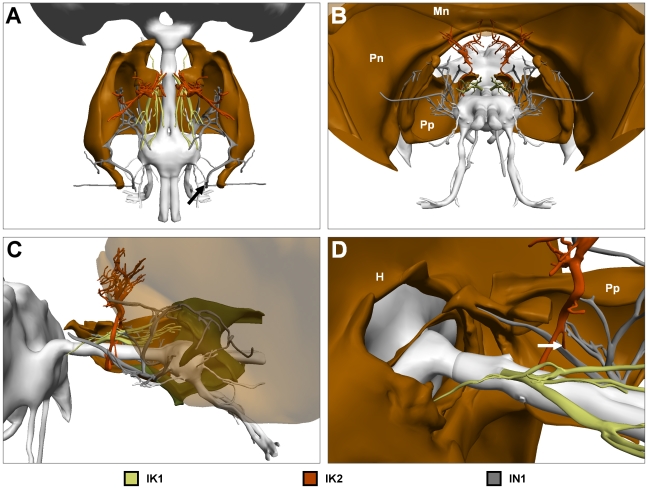
Ventral nerve cord (VNC) and nerves innervating the neck muscles; IK1, IK2 and IN1. (A) Dorsal view of brain, VNC and nerves. Arrow marks where IN1 meets IN6. (B) Posterodorsal view of nervous system with surrounding skeletal structures. Mesonotum shown translucent to expose full extent of IK2. (C) Lateral view of same with only right prospectus and mesonotum (translucent) shown. (D) Enlarged view of nerve cord exiting foramen magnum. For clarity only left IK1, and right IK2 and IN2 are shown. Arrow marks location where IK2 meets IN1. Colour codes and labels as in Fig. 1.

The ventral nerve cord projects through the occipital foramen below the tentorial bridge (Fig. 5D). The cord gives off two nerves (IK1 and IK2) before forming the prothoracic ganglion (Fig. 5A). Ten nerves emanate from the prothoracic ganglion, and these are numbered consecutively from anterior to posterior.

Innervation to all of the neck muscles is provided by motor neurons running through IK1 and IK2, and the first of the prothoracic nerves, IN1 (Fig. 5). IK1 innervates two ventrally located neck muscles. IK2 innervates many of the neck muscles originating from the pronotum or mesonotum. IN1 is the largest of the three cervical nerves, emanating in all directions to innervate several muscles of the neck and forelegs. A forward projecting branch of IN1 runs directly into the cervical apodeme of the propectus (Fig. 5D). It is densely packed with small diameter neurons that innervate mechanoreceptive hair cells, which detect movement between the occipital condyles of the head and the occipital processes of the propectuses. The second of the prothoracic nerves, IN2, runs to the floor of the propectus, where it presumably innervates a similar battery of mechanoreceptors registering movement between the propectuses and the basisternum. Similar prosternal organs have been described in *Calliphora* [15].

The forward mechanoreceptive branch of IN1 gives off a slender branch that unites with IK2 (arrow in Fig. 5D). Recurrent connections between the last nerve of one ganglion, and the first nerve of the succeeding ganglion is a feature of insect neural anatomy ([16]). Posteriorly, IN1 also unites with IN6 (Fig. 5A), as also occurs in locusts [16].

### Direct versus indirect head rotations

**Figure 6:**
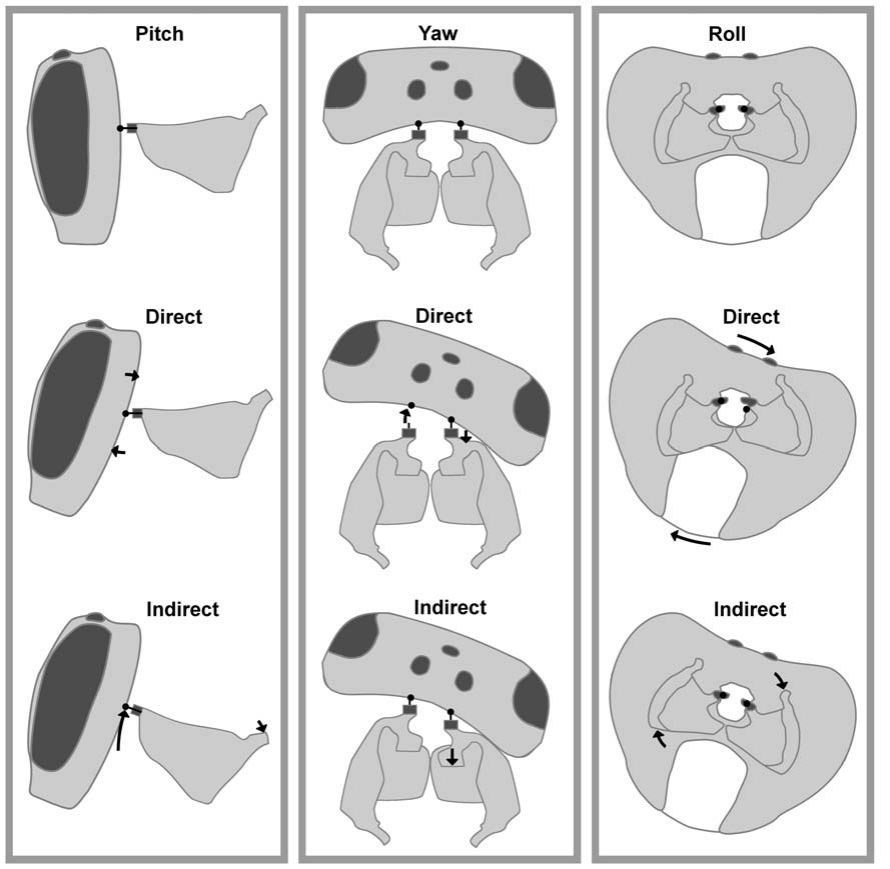
Schematic diagram illustrating direct and indirect head rotation around the three primary axes. Left: the head and propectus from the side in their natural position, after direct upward head pitch, and after indirect upward head pitch. Middle: the head and prospectus from above in their natural position, after direct rightward head yaw, and indirect rightward head yaw. Right: the head and prospectus as seen from the back in their natural position, after direct rightward head roll, and indirect rightward head roll. In all cases the head is rotated by 20°. Dark grey indicates the occipital processes, filled circles indicate the pivot points of the head.

Snodgrass [10] originally described two possible ways to move the head: direct and indirect. For direct movement, the head is moved relative to the supporting structures of the neck. Direct movement is elicited by muscles acting directly upon the head. For indirect movement, the head is not moved relative the supporting structures of the neck, but rather it is the supports themselves that are moved. Fig. 6 gives examples of direct and indirect head rotations.

Direct head rotation is complicated because the head is articulated about two pivot points. The pivots are located where the occipital condyles of the head meet the occipital processes of the propectuses. A consequence of articulation upon two pivot points is that the head is more easily rotated in pitch, than in roll or yaw. For roll or yaw to occur, the head must be released from its articulation with one of the pivot points.

Indirect head rotation is likely to be more effective in roll and yaw than in pitch. From consideration of Fig. 6, it is apparent that upwards pitch by indirect means is unlikely to occur because it would compress the gut, nerve tract and other soft tissues between the propectuses and pronotum. Head yaw can be achieved by protraction of one occipital process and retraction of the other. In reality it appears that the two propectuses are tightly coupled by a length of ligament-like tissue connecting the propectal condyle to the endosternum. Movement of one propectus induces movement of the endosternum, which in turn induces movement of the other propectus. However, rather than movement of one propectus relative to the other, indirect head yaw may be achieved by deformation of the propectus. Cross-sections of the propectuses showed that the sternal and pleural walls are very thin, suggesting considerable flexibility. Gently tugging the cervical apodemes of a dissected bee backward confirmed that the propectus is very flexible, allowing retraction of the occipital process while the base of the propectus remains stable.

Indirect roll can be achieved by rotation of the propectus-endosternum unit within the thorax. Indeed, roll rotation of the propectuses results in roll of the entire sternal base, which may then be supplemented by direct head roll.

Five direct muscles (muscles 40–44), and seven indirect muscles (muscles 45–51) [12], [13] have been described previously. Here we pay particular attention to the functions of the neck muscles, which we often find to be contentious with those attributed by Snodgrass [12] (see Table 1).

### Direct neck muscles—40 and 41

**Figure 7:**
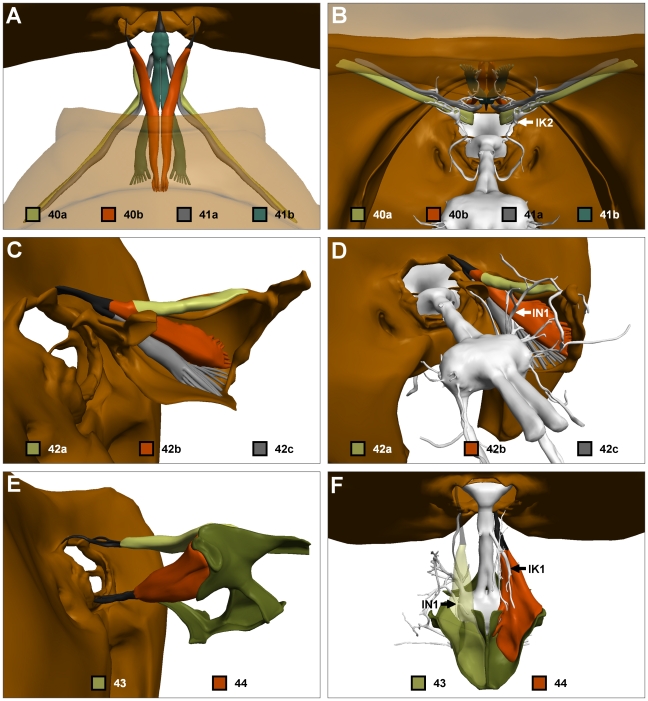
The direct neck muscles. (A) Dorsal view of muscles 40a–41b. Pronotum and mesonotum translucent. (B) Posterior view of same showing innervation by IK2. Mesonotum translucent. (C) Side view of right muscle 42a/b/c and right propectus. (D) Oblique posterodorsal view of same showing IN1 innervation. (E) Lateral view of muscles 43 and 44. (F) Dorsal view of left muscle 43 and right muscle 44 showing innervation by left IN1 and right IK1 respectively. Colour codes as in Fig. 1.

Both muscles 40 and 41 consist of two subunits (‘a’ and ‘b’) that are conjoined by a single tendon. The tendon of muscles 40a and 40b inserts laterally on the dorsal rim of the foramen magnum (Fig. 7A), or possibly fuses with the dorsal neck membrane; the tendon and membrane could not be differentiated here, and fusion of the tendon with the neck membrane has been described previously in locusts [16]. Muscle 40a runs laterally from the tendon to its origin on the prephragma of the mesonotum, while 40b runs medially from the tendon to its origin on the midline of the posterior pronotal inflection (Fig. 7A). The tendon of muscles 41a and 41b inserts on the centre of the dorsal rim of the occipital foramen. Muscle 41a runs laterally from the tendon to its origin on the prephragma, adjacent to 40a (Fig. 7A). Muscle 41b diverges laterally from the conjoined tendon, to attach to the medial aspect of the prephragma (Fig. 7A). Innervation to muscles 40 and 41 is given by IK2, which forms several points of contact with each subunit (Fig. 7B).

Snodgrass [12] described muscles 40 and 41 as levators of the head. Their attachments to the dorsal side of the neck foramen suggest a role in both upwards translation and upwards pitch of the head. The insertions of all subunits except 41b also suggest additional functions. Specifically, the lateral insertions of 40a suggest a role in generating head yaw. Muscle 40b, which shares the same tendon attachment to the head but projects dorsomedially rather than laterally, is likely to produce head roll to the contralateral side. Contraction of 41a may generate lateral translation of the head, though its dominant function is clearly head levation. As 40a and 41a curve underneath muscles 47 and 48, the direction in which the tendons are pulled depends on the rigidity of these muscles, with greater contraction of 47 and 48 progressively limiting movement in the yaw and roll axes respectively.

### Direct neck muscles—42

A large muscle composed of three branches that converge anteriorly to a common tendon that inserts on the tip of the supraforaminal apodeme of the head (Fig. 7C). Subunit 42a runs horizontal and attaches to the horizontal apodeme of the propectus. With some difficulty, subunits 42b and 42c could be dissociated in semi-thin sections by differences in staining intensity (42b being lighter). 42b and 42c form broad attachments on the pleural and sternal plates of the propectus respectively. All subunits are innervated by IN1 (Fig. 7D).

The insertion point of the tendon suggests that all subunits induce upwards pitch of the head. Contraction of 42a also generates substantial yaw in the ipsilateral direction, while 42b and 42c, which project further ventrally and laterally, are likely to generate strong head roll to the ipsilateral side: this is probably their dominant function.

### Direct neck muscles—43 and 44

Muscle 43 inserts just lateral of the supraforaminal apodeme of the head (Fig. 7E, 7F). Posteriorly, the muscle fibres bifurcate into two branches, one of which attaches to the lateral wall of the supraneural bridge of the endosternum, and the other on the medial, posterior and dorsal walls (Fig. 7F). The two branches of 43 could not be followed anteriorly, as the muscle fibres did not visibly differ in cross section. Both branches of 43 receive input from IN1 (Fig. 7F). The lateral attachment points and posterior projection of 43 suggests that this muscle is a powerful levator and yaw rotator of the head.

The largest of the direct neck muscles, muscle 44 attaches via a long tendon to the inner surfaces of the subforaminal cup of the head (Fig. 7E). The scoop shaped tendon encloses the anterior ends of the muscle fibres, which expand posteriorly before attaching to the endosternal wings and supraneural bridge of the endosternum (Fig. 7E, 7F). Muscle 44 consists of 5 discrete muscle subunits, each possessing anatomically distinct attachment points [14]. These subunits were not reconstructed here.

Innervation to 44 is provided solely by several branches of IK1 (Fig. 7F). Muscle 44 is the only direct neck muscle whose contraction can result in depression of the head, and this is its major function. Its off-midline insertion also suggests it may have a role in controlling head yaw.

### Indirect neck muscles—45

**Figure 8:**
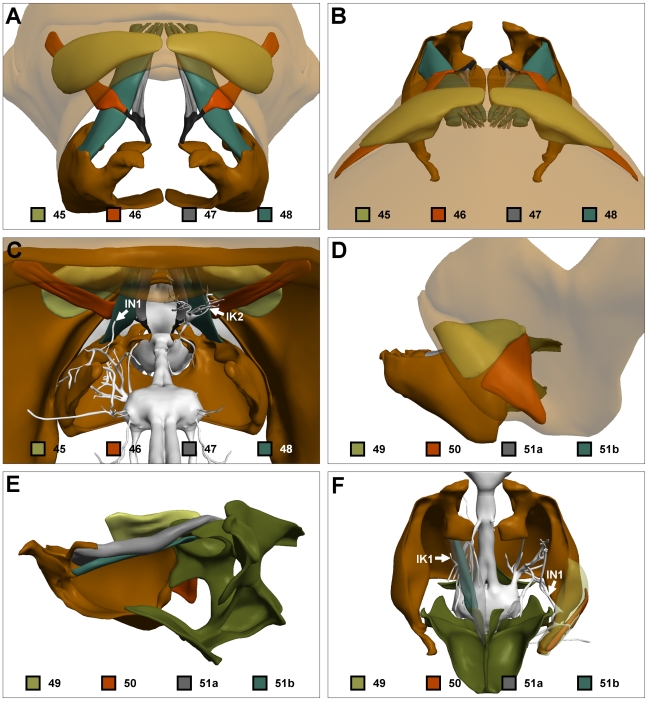
The indirect neck muscles. (A) Anterior view of muscles 45–48. Pronotum and mesonotum translucent. (B) Dorsal view of same. (C) Posterior view of same showing innervation by IN1 (left) and IK2 (right). Mesonotum translucent. (D) Side view of muscles 49–51. Pronotum translucent. (E) Side view of same with overlying pronotum and left propectus not shown. (F) Dorsal view of 49 and 50 (right), and 51a and 51b (left). Innervation by IN1 (right) and IK1 (left) also shown. 49 and 51a shown translucent to expose underlying 50 and 51b, respectively. Colour codes as in Fig. 1.

A stout muscle that connects the anterodorsal wall of the pronotum, to the mediolateral aspect of the prephragama (Fig. 8A–C). Innervation is provided by IK2. Snodgrass [12] considers this muscle as a depressor of the pronotum, or possibly retractor of the mesonotum. From our reconstruction it is unclear how contraction of this muscle could result in either of these functions. Rather it would seem that this muscle is a strong retractor of the pronotum. The very short length of the muscle (250 µm) suggests that its range of movement is very limited. Probably the major function of this muscle is simply to anchor the pronotum to the mesonotum.

### Indirect neck muscles—46, 47 and 48

Muscles 46 and 47 diverge from a common tendon that inserts on the cervical apodeme of the propectus. The tendon diverges, uniting laterally with muscle 46 which originates from the lateral walls of the pronotum, and medially with muscle 47, which originates from the posterior pronotal inflection (Fig. 8A–C). Markl [13] describes muscle 46 as being innervated by IK2, but was unable to determine which nerve innervates muscle 47. We find here that both 46 and 47 are innervated by IK2.

Muscle 48 is a large muscle that inserts broadly on the horizontal apodeme of the propectus. The muscle originates from the medial-most aspect of the posterior pronotal inflection (Fig. 8A–C). Muscles 47 and 48 run in very close association in this area, and it was difficult to distinguish the two fibre bundles. Muscle 48 is primarily innervated by IN1. Several branches of IK2 run in very close association with the external surface of muscle 48, but only one branch of IK2 appeared to breach the muscle surface and innervate the underlying fibres. Markl [13] also attributed innervation of 48 to both IN1 and IK2.

Snodgrass [12] did not describe the functions of these muscles, possibly because they are quite difficult to infer. Contraction of each of the three muscles results in levation of the occipital processes, suggesting possible roles in head pitch or upwards translation. However, the insertions of at least two of these muscles suggest that their dominant function is quite different. The medial insertion of 46 indicates that this muscle abducts and externally rotates the occipital process of the propectus, while the lateral insertion of 48 means that this muscle adducts and internally rotates the occipital process. Thus, these two muscles act antagonistically, possibly bringing about indirect head roll via rotation of the propectus-endosternum unit.

### Indirect neck muscles—49 and 50

Muscles 49 and 50 are closely associated and similar in appearance. Both muscles are fan shaped with broad basal origins located between the anterior pronotal inflection and the pleural wall of the pronotum. Tapering posteriorly, both muscles overly but do not attach to the pleural walls of the propectus; their insertion point is the apical tip of the pleural apophysis (Fig. 8D–F). Innervation to both muscles arises from IN1.

Snodgrass [12] describes both muscles as protractors of the propectus, as suggested by their posteriorly projecting insertions. We agree that both muscles protract the propectuses, but, we consider this to be the dominant function only for 49. Muscle 50 is a depressor, providing strong anchorage and support to the posterior bases of the propectuses, and possibly contributing to ipsilateral head roll.

### Indirect neck muscles—51

Snodgrass [12] and Markl [13] describe muscle 51 as a single muscle, while Schröter et al. [14] describe 51 as being composed of two closely related but anatomically distinct subunits (dorsal and ventral). Our results agree with the latter study, and in keeping with the established nomenclature we term the subunits 51a (dorsal) and 51b (ventral). Both subunits connect the cervical apodeme of the propectus to the supraneural apodeme of the endosternum (Fig. 8E–F). Anteriorly they are divided by a dense, forward projecting branch of IN1, posteriorly they are divided by the supraneural apodeme—51a attaching to the dorsal surface of the apodeme and 52b to the ventral surface (Fig. 8E). Both 51a and 51b are innervated by IK1.

Snodgrass [12] states that 51 is an adductor of the occipital processes. Here we consider the predominant function of 51a and 51b to be retraction of the occipital processes, with secondary adduction action. When muscle 51 contracts, the cervical apodemes are permitted to move inwards into the prothoracic cavity because the flexible nature of the propectal plates allows outward deformation of the propectuses. Thus muscle 51 generates indirect head yaw to the ipsilateral side.

### Indirect neck muscles—52

The last of the indirect neck muscles connects the base of the endosternum to the supraneural bridge of the mesothoracic endosternum, and is thus a retractor of the propectus [12]. This muscle lay beyond the posterior limits of our section series and was not reconstructed.

### The leg muscles

**Figure 9:**
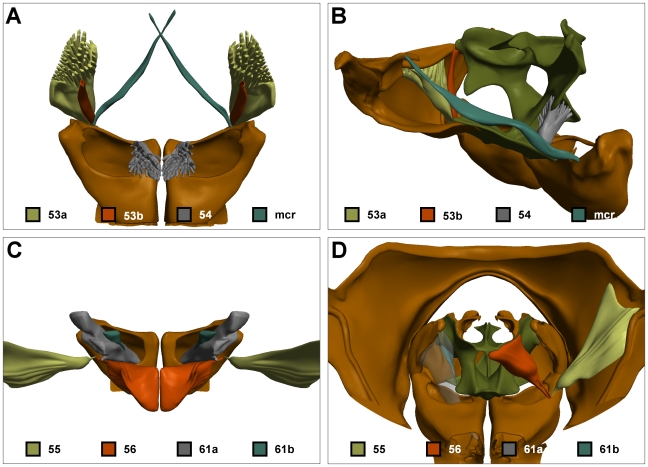
The leg muscles of the propectus, endosternum and pronotum. (A) Promoters of the fore coxae: dorsal view; (B) anterolateral view of right propectus, right 53a/b, left 54 and left mcr. (C) Remoters of the fore coxae: dorsal view; (D) Posterior view of right 55, right 56, left 61a (translucent) and left 61b. Colour codes as in Fig. 1.

Nine other muscles attach to the propectus, endosternum, or anterior pronotum. These are all muscles that are responsible for promoting (53a/b, 54, mcr) or remoting the fore coxae (55, 56) or trochanter (61a/b) (Fig. 9). While the dominant function of these muscels is to move the legs, it is possible that contraction of these muscles causes indirect head movement by depression of the propectus (53, 57, 61) or endosternum (54, 56), or flexion of the pronotum (55), thus linking movements of the forelegs to movements of the head. Certainly, these muscles provide substantial rigidity between the propectus, endosternum and coxae.

Here we also describe three muscles not described by Snodgrass [12]. Muscle 53b connects the anterolateral rims of the coxae, running adjacent to 53a, but separated by a sheath for most of its length before terminating on the lateral extremes of the supraneural bridge of the endosternum (not the propectus as for 53a). Muscle 61b merges with the muscle mass of 61a posteriorly, where it inserts on the anteromedial rims of the trochanter. Anteriorly, 61b diverges from 61a to attach on the underside of the endosternal wings rather than the pleural walls of the propectus. The fibres composing 61b are clearly smaller and of different type than those of 61a. The last of the undescribed muscles runs from the lateral extents of the cervical apodemes, crosses the midline of the body, and inserts anterolaterally on the contralateral coxae. Markl [13] also appears to have recognised these muscles, which he called the musculus cruciatus prothoracis.

## Discussion

We developed a comprehensive 3D digital atlas of the head-neck system of the honeybee. The model was generated from serial sections, because this was the only approach available to us capable of providing sufficient resolution to reconstruct the fine details of skeletal structures and nerve branches. The laborious nature of reconstruction from serial sections limited our sample size to one. Given that the anatomy of the honeybee neck described here agrees very closely with three earlier studies [12]–[14], we consider this atlas to be representative of the worker honeybee. Nevertheless, it is possible that the location of muscle attachment points, muscle masses, or cuticle rigidity at critical stress points varies between individuals of the same species. Such variability could lead to errors in biomechanical simulations based upon this work.

### Mechanics of skeletal morphology

The high resolution of our model, combined with the ability to independently show or hide individual muscles and skeletal structures greatly aided our understanding of the head-neck system. We concluded that the head articulates upon two pivot points (the occipital processes of the propectuses). This makes the head-neck joint of the bee especially well adapted for rotations in pitch (driven by direct muscles). Conversely, we concluded that mechanical and anatomical constraints make mechanisms using indirect muscles most effective for generating head roll and yaw (via deformation of the propectus).

The two levels of articulation, via direct and indirect muscles, bear similarities to the inner and outer rings of a gimbal. Rotation of the propectuses results in rotation of the head, which can then be rotated independently of the propectuses. Such a two-component gimbal system provides a method of allowing complex movements of the head in several axes. Gimbal-like mechanisms have also been proposed when describing the coxa-trochanter chain in the insect leg [17].

### Muscle morphology

Five direct neck muscles (muscles 40–44) and seven indirect neck muscles (muscles 45–51) have been classically described in the honeybee [12]. We consider this number to be an over simplification, because at least two of the direct neck muscles (muscles 40 and 41) clearly consist of two branches, each of which exert force in a different direction, and can therefore be considered a separate muscle.

Schröter et al. [14] also showed that neck muscles 44 and 51 are composed of five and two subunits respectively. Each subunit is anatomically separable, and each possesses unique attachment points on the endoskeleton and tendon. Some subunits may be differentiated on the basis of staining intensity or muscle fibre size, indicating that subunits may be composed of different fibre types [14], which is not unusual in invertebrates [18].

It was not deemed necessary to reconstruct subunits of muscles where all subunits exert force in a similar direction. However, observation of the sequential section series, and of the reconstructed surfaces clearly supported the conclusions of [14]. Additionally, we found here that muscle 43 bifurcates into two branches, suggesting that this muscle also consists of two distinct subunits. In this case both bundles of muscle fibres were of similar size and appearance.

### Muscle function

The accessibility of the model generated here allowed us to closely consider the functions of the neck muscles. For several of the neck muscles, we assigned different functions to those given previously [12] (summarised in Table 1). It is apparent that contraction of a single neck muscle may result in head movement in several degrees of freedom. Therefore, contraction of a neck muscle may cause a combination of translation, pitch, roll and yaw. As a result a single muscle may be useful in many different situations, and may receive a variety of visual and non-visual sensory inputs [14], [19]. The direction in which the head moves when a given neck muscle contracts is dependent on which other neck muscles are contracting at the same time. It follows that the most useful investigation of muscle function in such a system is not to consider the action of each muscle in isolation, but rather to consider which muscle interactions must occur to produce common head movements exhibited by bees. Correspondingly, we aim to use the 3D model as a tool for investigating synergistic muscle actions in a series of following studies. Similarly, electrical stimulation of combinations of muscles or groups of muscles, and high-speed video observations of head rotations in tethered bees may provide additional insights into the physiological actions and interactions of the neck muscles.

## Materials and Methods

### Animals

Foraging female bees (*Apis mellifera*) were obtained from hives at the Australian National University. A total of 7 samples were embedded. Of these, a single sample was selected for 3D reconstruction on the basis of minimal tissue damage, good infiltration of the embedding agent, and a natural head position.

### Histology

Cold anaesthetised bees were killed by gently cutting through the thorax just posterior to the first coxae, then submerging the head and prothorax in fixative (3.7% formaldehyde, 2.5% glutaraldehyde in 0.01 M phosphate buffered saline). The head capsule was exposed by removing the mouthparts and frons. Small incisions were made in the compound eyes and thorax to enhance fixative penetration. Samples were left in fixative at 4°C overnight. Samples were postfixed in 1% phosphate buffered osmium tetroxide, dehydrated through a graded acetone series, and embedded in an Araldite 502/Epon 812 resin. Fixation, dehydration and infiltration times were reduced by microwave radiation with concurrent vacuum pressure (Pelco BioWave 34700-230).

### 3D Reconstruction

**Figure 10:**
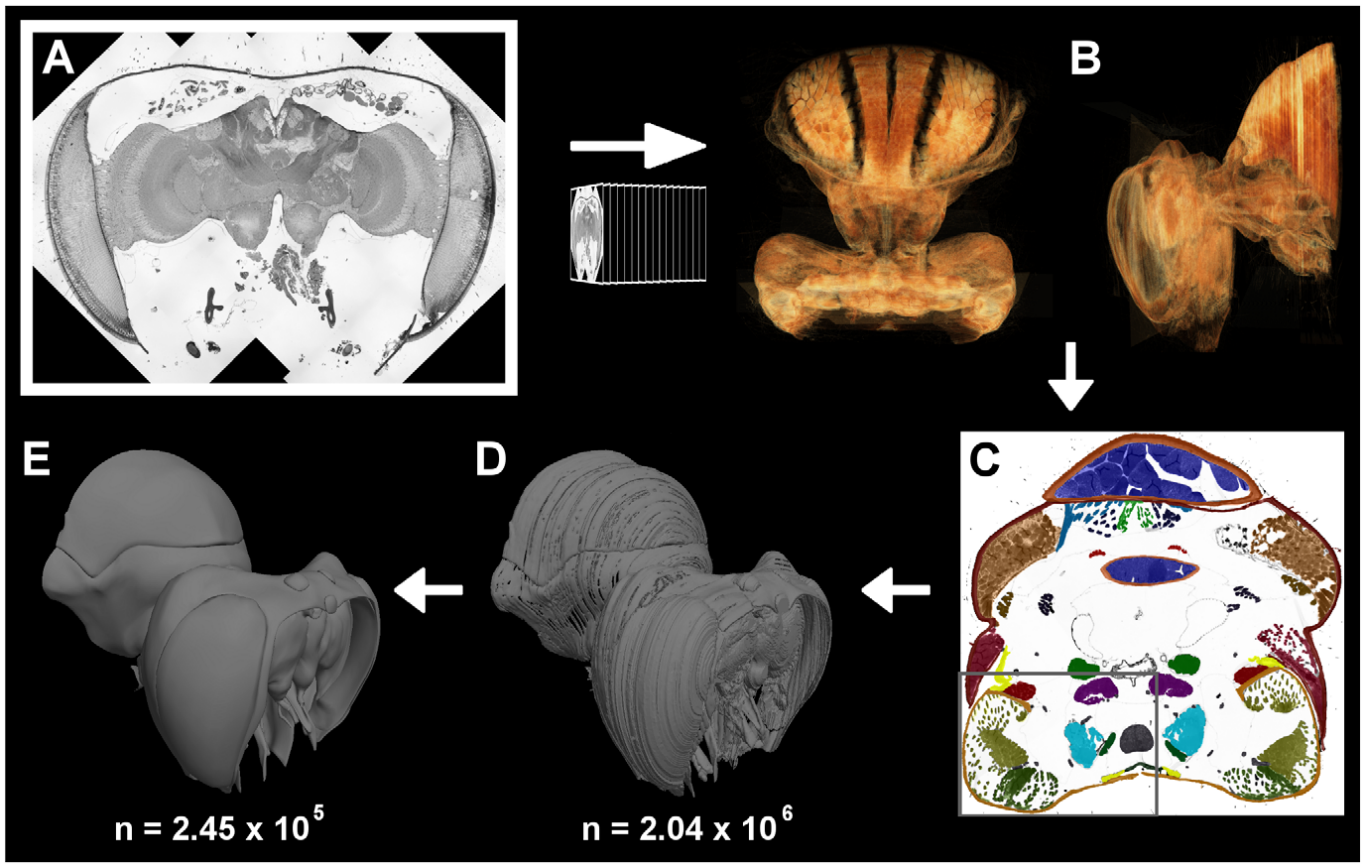
The steps involved in generation of the three-dimensional atlas. (A) Imaging: multiple images of each section were stitched together to create a single high-resolution image. Outline of multiple images are visible as white boundaries on the black background. (B) Alignment: images of each section were aligned relative to each other. Registration was quickly verified by volume rendering the image stack; the bee head and thorax are shown from the top (left) and side (right). (C) Segmentation: every structure of interest was manually outlined in each of the aligned sections. Different colours overlaid over this cross-section of the prothorax represent different structures. The boxed area illustrates the region shown in Fig. 11. (D) Model generation: mesh models were created from stacks of segmented images using Amira 3.1. (E) Redrawing: mesh models were greatly smoothed, simplified, and corrected for artefacts by manually redrawing using Silo 2.1. The approximate total number of polygons in each of the mesh models is indicated by *n*.

The specimen was serially sectioned with a diamond knife (Diatome, HistoJumbo) at 1 µm intervals on a Reichert-Jung ultramicrotome. Every tenth section was retained (resulting in a total of 304 sections), post-stained with toluidine blue and imaged on a Zeiss Axioplan 2 equipped with a Zeiss MRc camera. The large size of the sections relative to the high resolution required to resolve small nerve fibres necessitated imaging regions of each section individually, then stitching the multiple images together (Fig. 10A). This was facilitated using a semi-automated panoramic imaging module on the Zeiss Axioplan 2 (Zeiss AxioVision Panorama module). The outer boundaries of images were padded with black pixels, so that all resized images consisted of identical pixel dimensions (3171×3012 pixels) (Matlab, The MathWorks). The dimensions of the entire block were approximately 4.1×3.9×3.0 mm, with voxel (µm/pixel) dimensions of 1.289×1.289×10 (width×height×depth).

**Figure 11:**
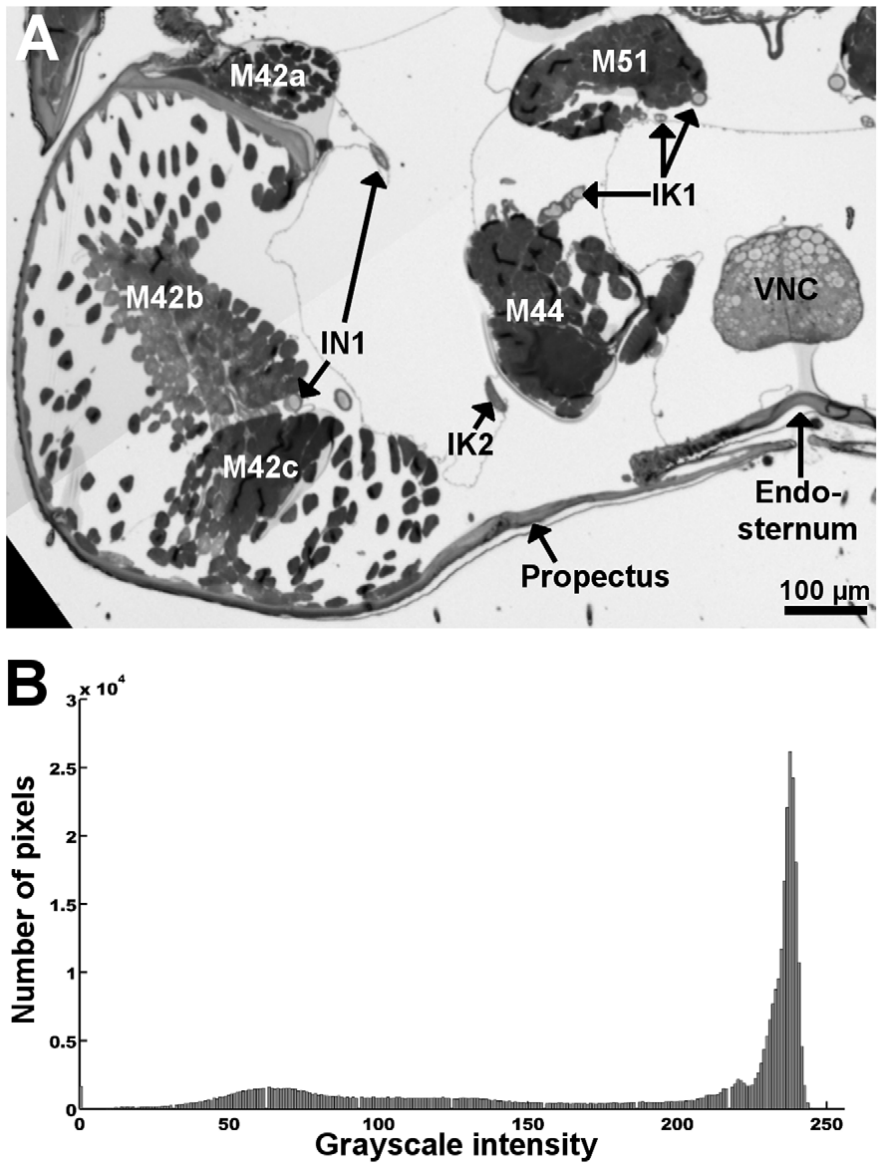
Representative image contrast. (A) Enlarged view of the marked region of section shown in Fig. 10C. The original image before segmentation is shown. Section was taken in a transverse plane, at a level near the anterior margin of the endosternum. The ventral direct neck muscles and their innervating nerves are visible. (B) Histogram of intensity values of the image shown in (A).

Images were imported into Amira 3.1 (Visage Imaging), and manually aligned such that they were deemed to have the best possible fit. Image registration could be quickly verified by volume rendering a down-sampled version of the entire image stack (Fig. 10B). After alignment, each image was segmented into discrete components by manually tracing every structure of interest (Fig. 10C). The segmentation processes was performed with little prior knowledge of the honeybee neck anatomy. Anatomical components reconstructed in the model were later identified by comparison to earlier studies of honeybee neck anatomy [12]–[14]. In general, image contrast was sufficient to allow segmentation of objects as small as 5–10 µm in diameter, such as fine nerve branches (Fig. 11). Contrast often varied little between the muscle fibres of individual muscles, making distinction of neighbouring muscles difficult. In these cases, the ability to follow muscle fibres by moving back and forth between adjacent serial sections greatly facilitated the segmentation process. Occasionally, regions of a section were obviously distorted and could not be reliably used for alignment or segmentation. In these cases, the region in question was segmented by interpolating between the two adjacent sections, or by using segmented regions from the previous section.

Following segmentation, Amira 3.1 was used to generate a mesh model of each structure of interest. Because of the large size and number of images, it was necessary to perform the segmentation and mesh generation process in blocks of approximately 50 images. The meshes generated by Amira (Fig. 10D) suffered a number of problems: discontinuities often occurred in clearly continuous structures; the boundary between sections appeared artificially step-like, due to the high voxel size in the z-dimension; the meshes had a very high polygon count. While the last two problems could be solved with smoothing and simplification algorithms, this often led to the loss of fine structural detail. To reduce polygon count and smooth surfaces without a loss of fine structure, the original meshes were imported into the 3D modelling software Silo (Nevercenter), and used as a base over which new meshes were manually drawn (Fig. 10E). To greatly facilitate this process, segmentation and redrawing were performed on one side of the bee, with the opposing side generated by mirror copying.

Individual fibres of some of the neck muscles were found to splay out at their origin. In these cases this was represented by drawing diverging muscle fibres. No attempt was made to accurately trace individual fibres.

## Supporting Information

Figure S1 Interactive three-dimensional atlas of the honeybee head-neck system. Requires viewing with Adobe Acrobat Reader 8.0 or greater in order to utilise 3D tools.
(21.44 MB PDF)
