# Supplementary figures and images for: A Three-Dimensional Atlas of the Honeybee Neck
Source: PLoS One. 2010 May 24;5(5):e10771. doi: 10.1371/journal.pone.0010771 (PMC2875396; doi:10.1371/journal.pone.0010771)

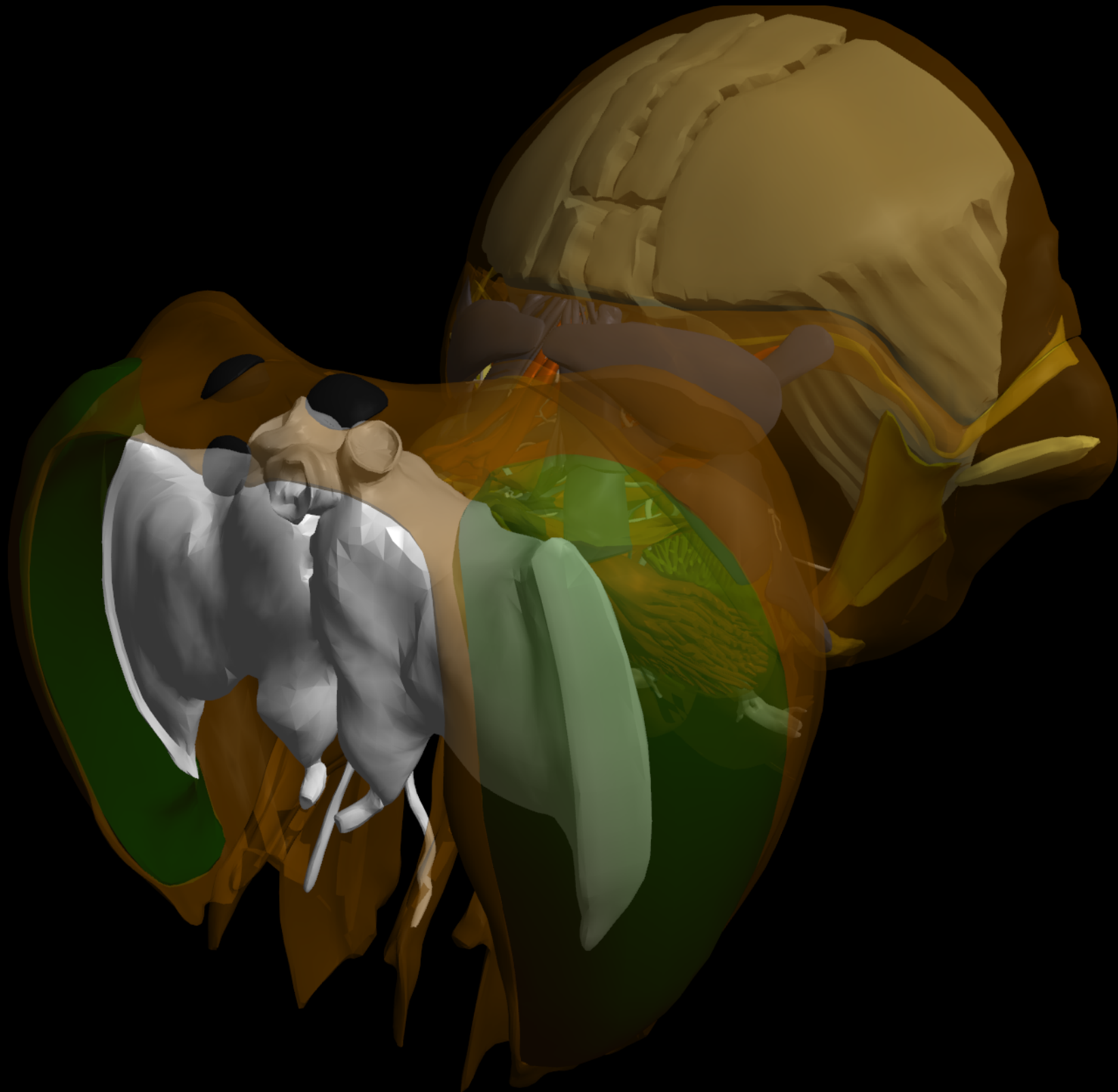

Supplement: Figure S1 — Interactive three-dimensional atlas of the honeybee head-neck system. Requires viewing with Adobe Acrobat Reader 8.0 or greater in order to utilise 3D tools. (21.44 MB PDF) [file pone.0010771.s001.pdf]
